# Veterinary Sanitary Science and Preventive Medicine

**Published:** 1884-04

**Authors:** Robert Ward

**Affiliations:** State Veterinarian of Maryland


### ART. XII.—VETERINARY SANITARY SCIENCE AND PREVENTIVE MEDICINE.

BY DR. ROBERT WARD, F. R. C. V. S.

*State Veterinarian of Maryland.*

PROPHYLACTIC measures very properly belong to the domain of the advanced or modern Veterinarian, and it should be the paramount duty of all states to exercise the authority they possess in this direction.

State Medicine is that branch of medical science which grasps the well being of the masses, and therefore becomes a most important item in the commonwealth of nations.

Still in our day, this important branch with that of comparative pathology are held in abeyance for future generations to develop, as any new suggestion in this direction is pooh-poohed by those in authority, and treated as an *innovation*, however correct the *data* may be.

Experiments on living animals, however, are instituted with unabated vigor, to ascertain the *fact* of the potency of certain specific materies morbi and its period and phases of incubation, the data of which has been given by the most reliable authority of modern times, and the *fact* handed down to us by these learned *savants*.

Time goes on, and with it these diseases under scientific experiment or investigation, no practical benefit accruing, no *quid pro quo*; and certain it is that, unless the medical press of this country comes to the rescue, this condition of things may go on forever, and at the same time the dreaded "pleuro pneumonia contagiosa,"—a term which has haunted the public and its press, *ad nauseam*.

It must not however be surmised for a moment that I am reckless on the etiology of diseases, on the other hand I opine it to be of the highest importance, and I do desire to see an earlier recognition and development of preventive medicine than at present obtains.

The pathology of fatal diseases communicable by *contagia* alone, has opened up a field of scientific research which, in Europe, has taken a wide range. The microscope has clearly defined the organic life which is the active aggressive agent in disturbing the equilibrium of health, and the organisms have been so cultivated that, whilst they retain certain characteristics, the *active principle* is so modified that by inoculation with the virus thus treated, an immunity from the morbid influences of the disease germs is obtained without any visible or particular disturbance of the health of the subject so operated upon.

As far as scientists need go in this particular direction, the experiments of Pasteur, Chauveau and Toussaint, are sufficiently demonstrative, and those who may not at this present day grasp the *facts* submitted to their ken, may safely be left behind on the road to scientific research, till their state becomes equivocal, when they can follow after us, who have gone on in the van.

As a preventive medicine—inoculation has, by recorded practical facts in medical history, proved itself to be a most valuable prophylactic in pleuro-pneumonia, and I do unhesitatingly assert that, for our city dairies, it is the modus operandi that should be generally adopted, that is to say, in those cities wherein the trouble has so long existed. Not only should the dairy cows be submitted to the operation, but no cow should be permitted to enter until the operation has been successfully performed.

It must not be supposed by the stock-owners that inoculation does produce the disease with all its characteristics. The pathological lesions attending the operation clearly demonstrate that it is not so, but as *small pox* is not developed by *vaccine*, so also pleuro-pneumonia is not developed by its modified virus; by it, however, a systematic shock is obtained which renders the system non-susceptible to the morbid influences of the disease germ.

That this is so, Europe and the English colonies have evidence demonstrative, the result of sound, practical experience, which is open to all the world.

It has been said by some skeptics that inoculation harbors the infective element for a remote period, now what is the established evidence of experience against this?

The evidence adduced at the International Veterinary Congress held at Brussels in October last, goes to this effect:

The value of inoculation as a prophylactic measure has been generally recognized, and the question as to whether it might perpetuate the disease by healthy animals becoming infected by those inoculated, appears to be decided in the negative.

And again it was held by a vast majority that it was purely a contagious malady in its origin and its propagation, the evidence being against development 'de novo' or transformation of germs.

No sense of my duty as a State Veterinary Officer has urged me to write this matter into notice, and because I have been asked to express my opinion, which is opposed, I find, to some of my colleagues in office, I distinctly say that the value of inoculation, under certain circumstances, as a *preventive medicine* in contagious pleuro-pneumonia cannot be over estimated.

The operation properly performed is not a simple surgical operation, but a really scientific one, and needs some care and careful manipulation. The rude method of slitting the tail and tying a piece of diseased lung on the wound is as absurd as it is barbarous, and doubtless the after consequences have brought the operation into bad repute.

At some future time, should your readers desire it, I will enter into details concerning the modus operandi for successful inoculation, and I trust some good may follow from the remarks I have made, which are the result of experience and honest conviction.

